# Rapid on-site diagnosis of canine giardiosis: time *versus* performance

**DOI:** 10.1186/s13071-020-04422-6

**Published:** 2020-11-02

**Authors:** Isaia Symeonidou, Athanasios Ι. Gelasakis, Androulla N. Miliotou, Athanasios Angelou, Konstantinos V. Arsenopoulos, Sofia Loukeri, Elias Papadopoulos

**Affiliations:** 1 Laboratory of Parasitology and Parasitic Diseases, School of Veterinary Medicine, Faculty of Health Sciences, Aristotle University of Thessaloniki, 54124 Thessaloniki, GR Greece; 2 Laboratory of Anatomy and Physiology of Farm Animals, Department of Animal Science, School of Animal Biosciences, Iera Odos str. 75, 11855 Athens, GR Greece; 3 Laboratory of Pharmacology, School of Pharmacy, Aristotle University of Thessaloniki, 54124 Thessaloniki, GR Greece; 4 Medical Department Virbac, 13ème Rue, 06511 Carros, France

**Keywords:** *Giardia* spp., Dogs, Diagnosis, Speed^TM^*Giardia*, Microscopy, PCR

## Abstract

**Background:**

Infections by protozoans of the genus *Giardia* are a common cause of diarrhea in dogs. Canine giardiosis constitutes a disease with a zoonotic potential; however, it is often underestimated due to its challenging diagnosis. The objective of the study was to assess the diagnostic performance of an immunochromatographic strip test (Speed^TM^ *Giardia*, Virbac, France) comparing it with microscopy (zinc sulfate flotation) by utilizing the combination of an enzyme immunoassay (ProSpecT^TM^ *Giardia* EZ Microplate Assay, Oxoid Ltd., UK) and the PCR as the gold standard. A positive result in both ELISA and PCR was set as the gold standard.

**Methods:**

Initially, fecal samples from dogs with clinical signs compatible with giardiosis were tested with the Speed^TM^ *Giardia* test and separated into two groups of 50 samples each: group A (positive) and group B (negative). Thereafter, all samples were examined by zinc sulfate centrifugal flotation technique and assayed by the ProSpecT^TM^ *Giardia* Microplate Assay and PCR. The performance of the Speed^TM^ *Giardia* and zinc sulfate centrifugal flotation tests were calculated estimating sensitivity, specificity, and positive and negative likelihood ratio; the chi-square and McNemar tests were used for the comparison of the two methods.

**Results:**

*Giardia* cysts were not detected by microscopy in 16 out of the 50 samples (32%) of group A and in none of group B samples. Eight out of 50 samples in group B (16%) were tested positive both with the ProSpecT^TM^ *Giardia* Microplate Assay and PCR. Fecal examination with the Speed^TM^ *Giardia* test was more sensitive (86.2%) than the parasitological method (58.6%, *P* < 0.001) while the specificity of both methods was 100%.

**Conclusions:**

The Speed^TM^ *Giardia* test is an easy-to-perform diagnostic method for the detection of *Giardia* spp., which can increase laboratory efficiency by reducing time and cost and decrease underdiagnosis of *Giardia* spp. infections. This immunochromatographic strip test may be routinely exploited when a rapid and reliable diagnosis is required, other diagnostic techniques are unavailable and microscopy expertise is inefficient. In negative dogs with compatible clinical signs of giardiosis, it is recommended either to repeat the exam or proceed with further ELISA and PCR testing.
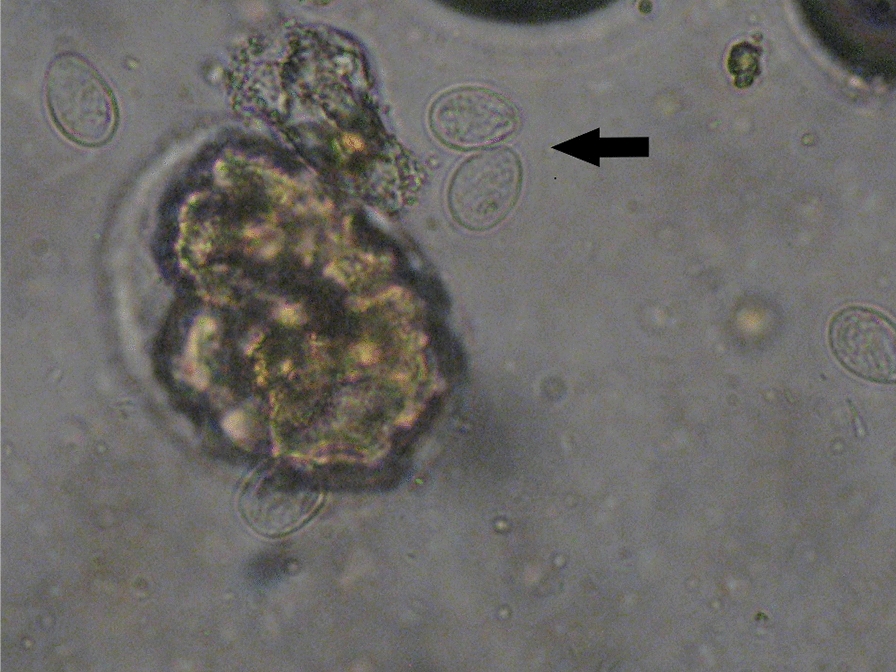

## Background

*Giardia* spp. are flagellated protozoans that colonize the duodenum of many mammals, including dogs and humans [1]. Among the several species of *Giardia*, only *Giardia duodenalis* (syn. *G. lamblia*, *G. intestinalis*) has been recovered from both the aforementioned mammals, hence being regarded as potentially zoonotic [2–5]. The transmission chain includes the defecation of *Giardia* spp. cysts, which are infective immediately after excretion, the contamination of feeds and water and the infection *via* the fecal-oral route [6]. *Giardia* spp. infections may impair dogs’ health and welfare resulting in a wide spectrum of clinical manifestations, such as diarrhea, bloating, abdominal discomfort, weight loss, malabsorption, growth retardation and sometimes even death, especially in puppies [7–9]. The occurrence of disease depends on many factors, such as the *Giardia* strain virulence, the parasite burden, the age and immunity of the host, whereas many animals remain sub-clinically infected and act as reservoirs of the parasite.

*Giardia duodenalis* contains at least eight distinct genetic assemblages (A-H) as demonstrated by molecular typing methods [10, 11]. Assemblages A and B have been reported in many mammals, including humans [12–15] and dogs [16, 17] and have a zoonotic potential which renders them of great public health concern [9, 18–20]. Currently, there is cumulative evidence that dogs act as a source of contamination for humans and pose a risk especially for pet owners and shelter staff [21, 22]. Also, giardiosis outbreaks due to contaminated drinking water and food have been reported [23–26].

Giardiosis is a common parasitosis even in the well-cared dog populations [27]. Relatively high prevalence of canine *Giardia* infection has been reported in many European countries by employing microscopy (28.5% for Belgium, 27.5% for France, 25.9% for Italy, 25.1% for Spain, 24.6% for the Netherlands, 23.8% for Germany, and 14.6% for the UK) [28]. In a recent large-scale study conducted in Greece, *Giardia* spp. were detected by microscopy following zinc sulfate flotation in 9.5% of the sampled dogs with no apparent clinical signs [29]. Studies employing immunological and molecular assays have reported much higher prevalence than microscopy [28], indicating that *Giardia* spp. infections may remain underdiagnosed when microscopy is the selected exam. In any case, the infection level is considerably higher in young animals [30], while it is up to 100% in dogs living in kennels or shelters [31], due to overcrowding and inadequate hygiene conditions [32].

The well-documented pathogenicity of *Giardia* spp. both for humans and animals, coupled with the high prevalence of giardiosis underpin the demand for universally accepted diagnostic tests and protocols for the early and accurate diagnosis of this disease. Therefore, the development of valid and cost-effective assays is essential for the surveillance of the disease and the evidence-based planning for its control (preventive measures and treatment) [33].

Nowadays, a variety of diagnostic methods for canine giardiosis are available. Among them, microscopy following zinc sulfate flotation, and immunoassay methods such as the direct fluorescent antibody (DFA) tests, which detect intact parasites [34], and the immunofluorescence antibody (IFA) microscopy, which detects epitopes of cysts [35], are commonly used. Other immunoassays include the enzyme-linked immunosorbent assays (ELISA) and the immunochromatographic lateral-flow tests, also known as rapid diagnostic tests (RDT), and detect soluble coproantigens of the parasite [36–39]. RDT are qualitative, commercially available enzyme immunoassays, which have become popular diagnostic tools for practitioners [36, 38]. Finally, molecular techniques such as the polymerase chain reaction (PCR) have also been developed for the detection of *Giardia* spp. [6, 40, 41]. All the aforementioned methods have both advantages and limitations and the selection of the suitable diagnostic tests in practice is mostly dependent on their performance, the availability of laboratory infrastructures and equipment, the personnel expertise, as well as their quickness and cost-effectiveness [42, 43].

The aim of this study was to evaluate the performance of the Speed^TM^ *Giardia* test (Virbac, Carros, France), a rapid immunochromatographic lateral-flow test for the detection of *Giardia* spp. in canine fecal samples and to compare it with microscopy, using the combination of enzyme immunoassay ProSpecT^TM^ *Giardia* Microplate Assay (Oxoid Ltd., Hampshire, UK) and PCR as gold standard.

## Methods

### Dog population and fecal sample collection

A total of 100 dogs with diarrhea (the main clinical sign of giardiosis) were included in the study. Canine fecal samples were collected from local animal shelters and veterinary clinics in northern Greece, between February and June 2018. Sex was almost evenly distributed (52 male and 48 female dogs) while all dogs were older than 6 months. None of the examined animals received any antiparasitic treatment at least 3 months prior to inclusion. From each individual dog, a fecal sample was collected either immediately after spontaneous elimination or fresh from kennel grounds avoiding contamination. Samples were placed individually in plastic containers, labelled with consecutive numbers, stored at 2–6 °C, transferred to the Laboratory of Parasitology and Parasitic Diseases of the School of Veterinary Medicine, Thessaloniki, Greece, and processed within 1 day.

### Rapid diagnostic test (RDT)

Initially, the Speed^TM^ *Giardia* assay was performed according to the manufacturer’s instructions. In brief, one spoonful of each labelled fecal sample was added to the buffer diluent in a corresponding vial, which was closed and shaken to homogenize. The solution was allowed to sediment for 3 min. The strip was gently plunged into the solution in the direction indicated by the arrow and allowed to stand for 1 min, without immersing the central reactive zone in the solution. Thereafter, it was removed and placed on a flat, horizontal surface. The liquid was left to migrate and the results were read after 5 min. The test was valid when a blue control band appeared. The test was considered as positive when a red band appeared at the *Giardia* test window along with the blue control band. Any red colouration of the test band regardless of colour gradation was interpreted as a positive result. Two groups of animals emerged, each consisting of 50 dogs. Group A consisted of 50 *Giardia*-positive dogs and group B consisted of 50 *Giardia*-negative dogs.

### Microscopy following zinc sulfate flotation

All fecal samples were examined by qualitative flotation with zinc sulphate (ZnSO_4_ 33.2%, specific weight 1.3) [44–46]. In detail, 1 g of feces was diluted with water, passed through a sieve (No. 150) into a centrifuge tube and centrifuged at 200× *g* for 3 min. The supernatant was discarded and zinc sulphate solution was added to the sediment, which was then completely diluted. Zinc sulphate solution was added to the tube so as to form a crescent and a coverslip was placed on top of it. Following centrifugation at 150× *g* for 1 min, the coverslip was carefully removed and placed on a microscope slide. Microscopic examination was carried out by the same experienced parasitologist. Identification of *Giardia* spp. cysts was based on morphological characteristics [45, 47]. A dog was considered positive if at least one cyst was observed.

#### ELISA

A copro-antigen ELISA was performed using the ProSpecT^TM^ *Giardia* EZ Microplate Assay (Oxoid Ltd.) for all the samples according to the manufacturer’s instructions. This immunoassay uses a monoclonal antibody for the qualitative detection of *Giardia* specific antigen 65 (GSA 65) in aqueous extracts of fecal specimens.

### DNA extraction

Genomic DNA was extracted directly from all preserved *Giardia*-positive and *Giardia*-negative fecal samples using QIAmp**®** Fast DNA Stool Mini Kit (Qiagen, Hilden, Germany) according to the manufacturer’s protocol for isolating DNA for pathogen detection. To maximize cyst lysis, an initial step of three freeze-thaw cycles (heating at 80 °C water bath for 5 min, followed by freezing at -20 °C for 5 min) was incorporated in the protocol as proposed by Tan et al. [1]. The extracted DNA was eluted in 50 μl of elution buffer, and all the eluates were stored at -20 °C until further molecular analyses.

### PCR amplification of the *18S* rRNA gene

A region of the *18S* ribosomal RNA gene was amplified by using a forward primer RH11 (5′-CAT CCG GTC GAT CCT GCC-3′) and a reverse primer RH4 (5′-AGT CGA ACC CTG ATT CTC CGC CAG G-3′) as described by Hopkins et al. [40]. The predictive amplification fragment was 292 bp. All PCRs were performed in a total volume of 25 μl containing 4 μl DEPC (diethyl pyrocarbonate) water, 2 μl of each primer RH11/RH4 (50 μmol/l), 2.5 μl One *Taq* high GC enhancer, 12.5 μl One *Taq* 2× master mix with GC Buffer (M0483S; New England BioLabs Inc., Hitchin, UK) and 2 μl DNA template. All reaction components were assembled on ice. The thermocycler program consisted of an initial denaturation of 96 °C for 4 min, followed by a set of 35 cycles, each consisting of 20 s at 96 °C, 20 s for annealing at 59 °C, 30 s at 72 °C, followed by a final extension step at 72 °C for 7 min. Along with the samples a negative control (doubled distilled water) and a positive control (genomic DNA from a fecal sample positive in all other three tests) were tested for each reaction. All amplification products were submitted to 1.5% ethidium bromide-stained agarose gel electrophoresis. The obtained gel images were recorded with a CCD camera under UV light and visualized with the MiniBis Pro gel documentation system (DNR BioImaging systems, Neve Yamin, Israel).

### Statistical analyses

Initially, data were recorded in a specially designed Microsoft Excel spreadsheet. In the subsequent statistical analyses, accuracy [(true positive + true negative)/(true positive + true negative + false positive + false negative)], sensitivity [Sn, true positive/(true positive + false negative)], specificity [Sp, true negative/(true negative + false positive)], positive likelihood ratio [LR+, Sn/(1−Sp)] and negative likelihood ratio [LR−, (1−Sn)/Sp] were calculated from the 2 × 2 contingency tables of the studied methods using chi-square test in SPSS 23. McNemar test was used for the comparison between Speed^TM^ *Giardia* test and microscopy. A positive result in both ELISA and PCR was chosen as the gold standard.

## Results

**Fig. 1:**
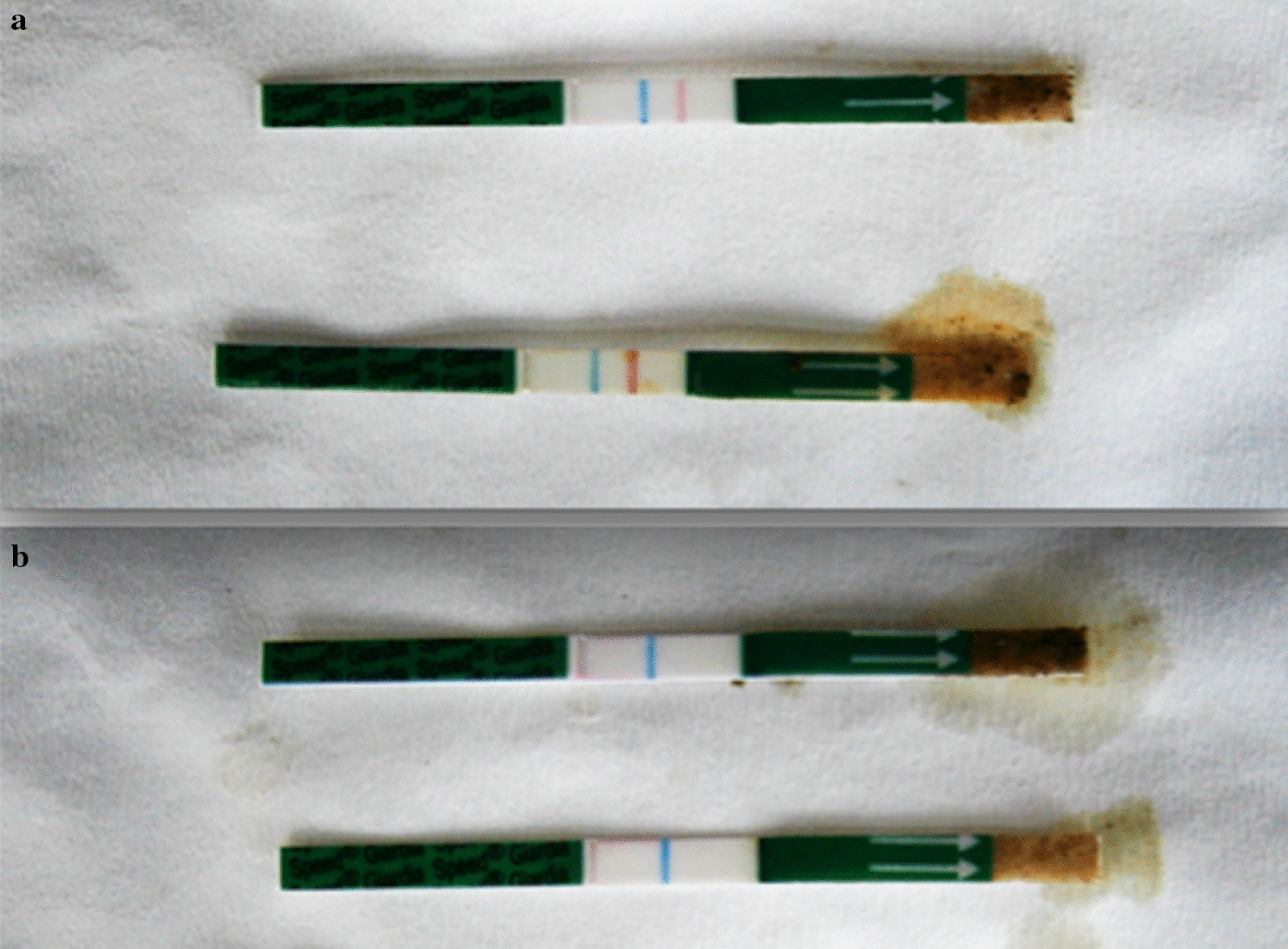
**a** Samples testing positive with the Speed^TM^ *Giardia* test (blue line: control band, red line: positive samples). **b** Samples testing negative with the Speed^TM^ *Giardia* test (blue line: control band, no other line was detected: negative samples)

**Fig. 2:**
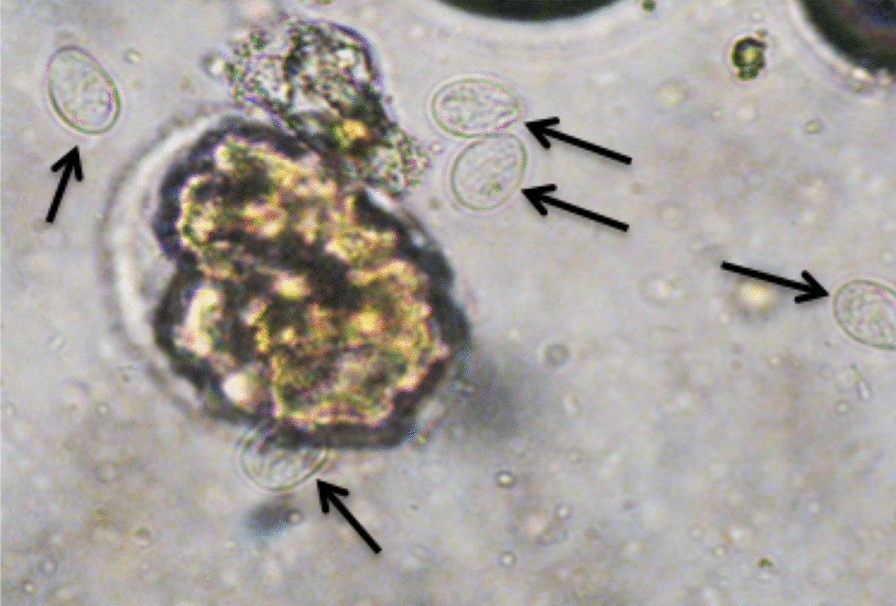
*Giardia* spp. cysts (arrow) as seen with microscopy (400× magnification) following the zinc sulfate flotation technique

Out of the 50 samples of group A (positive with the Speed^TM^ *Giardia* test, Fig. 1a), microscopy confirmed 34 (68%) positive for *Giardia* spp. (Fig. 2). In the remaining 16 (32%) samples of group A *Giardia* cysts were not detected during microscopy. On the contrary, results from the microscopic examination of the 50 samples of group B (negative with Speed^TM^ *Giardia* test, Fig. 1b) were in agreement (100%) with the Speed^TM^ *Giardia* test results. All samples in group A were ELISA-positive, while 15 out of 50 samples (30%) in group B were also ELISA-positive. Eight out of the 15 ELISA-positive samples were also PCR-positive, whereas the remaining 7 were PCR-negative (Table 1).

**Table 1:** 2 × 2 contingency tables of the four techniques used for the diagnosis of *Giardia* spp. infection

	ELISA and PCR combined		Total
	Negative	Positive	
Speed^TM^ *Giardia*			
Negative	42	8	50
Positive	0	50	50
Microscopy			
Negative	42	24	66
Positive	0	34	34
ELISA			
Negative	35	0	35
Positive	7	58	65
PCR			
Negative	28	0	28
Positive	14	58	72
Total	42	58	100

**Fig. 3:**
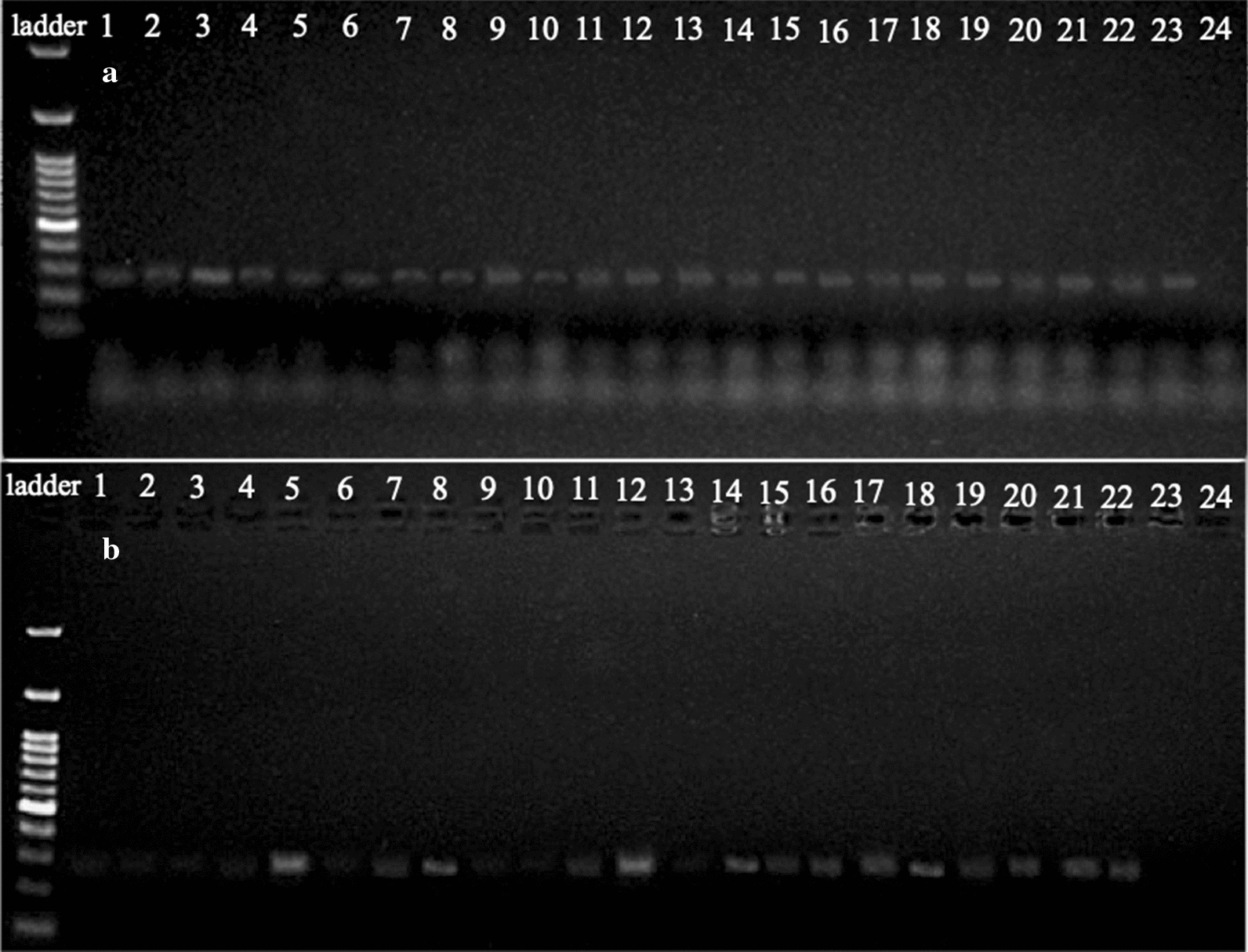
Positive and negative samples with PCR from the examined groups of dogs. PCR products analysed by electrophoresis on 1.5% agarose gel, stained with ethidium bromide and visualized on a UV transilluminator. The band of 292 bp corresponds to the amplified product of the *18S* rRNA gene. **a** Lane L: DNA ladder; Lanes 1–23: positive samples from group A; Lane 24: control. **b** Lane L: DNA ladder; Lanes 5, 7, 8, 11, 12, 14–22: positive samples from group B; Lanes 1–4, 6, 9, 10, 13, 23: negative samples from group B; Lane 24: control

PCR amplifications of the *18S* rRNA gene of *Giardia* spp. were consistent with the expected size (292 bp), without non-specific bands. Samples from group A were all PCR-positive (Fig. 3a). Twenty-two out of the 50 samples from group B (44%) tested positive by PCR (Fig. 3b). Fourteen out of these 22 samples were ELISA-negative (Table 1).

Sensitivity, specificity, accuracy, positive and negative likelihood ratios for the four diagnostic tests are summarized in Table 2. Fecal examination with the Speed^TM^ *Giardia* assay was more sensitive (86.2%) than microscopy (58.6%, McNemar test, *P* < 0.001) while the specificity of both methods was 100%.

**Table 2:** Sensitivity, specificity, accuracy, positive likelihood ratio and negative likelihood ratio of the four studied methods. The combination of ELISA and PCR was used as reference method

Method	Accuracy (%)	Sn (%)	Sp (%)	LR+	LR−
Speed^TM^ *Giardia*	92.0	86.2	100.0	Infinity	0.1
Microscopy	76.0	58.6	100.0	Infinity	0.4
ELISA	93.0	100.0	83.3	6.0	0.00
PCR	86.0	100.0	66.7	3.0	0.00

*Abbreviations*: Sn, sensitivity; Sp, specificity; LR+, positive likelihood ratio; LR−, negative likelihood ratio

## Discussion

The objective of this study was to evaluate the diagnostic performance (sensitivity and specificity) of the Speed^TM^ *Giardia* method by comparing it with microscopy using the combination of ELISA and PCR.

Speed^TM^ *Giardia* assay is a commercially available RDT. This test allows the detection of specific soluble antigens of *Giardia* spp. in preserved canine fecal samples [36, 39]. Up to date, a number of commercial RDT have been developed for the detection of *G. duodenalis* coproantigens in dogs [34, 37] and they have become increasingly popular [38]. Overall, several studies have evaluated the methods for diagnosing canine *Giardia* spp. infections [48–54], but, none of them so far has assessed simultaneously the four tests employed here and compared the Speed^TM^ *Giardia* test with microscopy. In the present study, a positive ELISA and PCR was defined as the gold standard. To set the gold standard, the combination of the two methods was preferred due to the high sensitivity and specificity of both methods and the lack of a validated and universally accepted gold standard for the diagnosis of giardiosis [38, 52, 54]. In detail and according to the international literature, PCR assays exhibit high sensitivity and specificity [52, 55–57], whereas the reported sensitivities and specificities of commercially available ELISA range from 94 to 97% and from 99 to 100%, respectively [37, 58, 59]. ELISA use antibodies for the qualitative detection of *Giardia*-specific antigens in fecal specimens [36, 60]. The ProSpecT^TM^ *Giardia* EZ Microplate Assay was selected as being one of the most reliable ELISA [61, 62]. It is a fast assay, as it requires the fewest washing steps, it provides a more efficient interpretation of the results and also has a very high specificity and positive predictive values (98–100%) as well as the highest sensitivity (96–98%) among the other commercially available ELISA [61, 63].

The Speed^TM^ *Giardia* test displayed higher sensitivity (86.2%) compared to microscopy (58.6%, *P* < 0.001), being in accordance with numerous other studies, which have demonstrated that various different commercial RDT were more sensitive than microscopy [38, 50–52, 54, 64–66]. It is indeed expected that tests based on antigen detection, such as the RDT, to be more sensitive than microscopy and to at least approach the PCR results, as highlighted by McGlade et al. [55]. Although microscopy following zinc sulfate flotation for the recovery of *Giardia* spp. cysts is a commonly used method for the diagnosis of giardiosis, it has low sensitivity, as is the case of the present study (58.6%). This can be attributed to the intermittency of excretion of this protozoon in canine feces [67, 68]. Given this excretion pattern of *Giardia* cysts, a single coprological examination could partially explain the occasional inexistence of cysts during microscopy in the present study, as the fecal samples were collected and examined only once. Moreover, a low concentration of shedding cysts (not detectable with microscopy), and/or collapsed cysts (cysts with different density which are not able to float and be identified) may be associated with the low sensitivity recorded for microscopy [69]. Based on the above, it is evident that a single negative result may not definitely determine the presence or absence of *Giardia* spp. in examined dogs and therefore re-examination of fecal samples is necessary to increase the sensitivity of the method [48, 70, 71]. The demand for repeated testing renders microscopy a time-consuming and labor-strenuous method. However, poor performance of microscopy has been reported even in cases where consecutive samples were pooled and tested [66]. This can be justified due to the fact that even at the peak of cyst excretion, the accurate identification of *Giardia* cysts still requires personnel with a high level of expertise. *Giardia* cysts can be easily misdiagnosed because of their small size (8–12 μm × 7–10 μm) and their resemblance to plant remnants, yeasts and debris, which are common in fecal samples [50]. Consequently, the Speed^TM^ *Giardia* test provides an alternative assay to overcome diagnostic challenges in clinical practice. This RDT detects excreted antigens and thus cyst identification is no longer required, overcoming the major drawback of microscopy [72]. According to relevant research findings, it is recommended to use centrifugal fecal flotation in conjunction with an immunoassay for increasing the sensitivity of diagnosing *Giardia* spp. infections in veterinary practices [68, 69].

Nevertheless, in our study eight out of the 50 samples (16%) of group B (negative by the Speed^TM^ *Giardia* assay) were tested positive both with the ProSpecT^TM^ *Giardia* EZ Microplate Assay and PCR. This lower sensitivity of the RDT (86.2%) compared to that of the two methods combined is an expected finding, as the combination of the two methods was considered the gold standard in this experimental design. This is in accordance with other studies where RDT false negative results were observed in cases of low parasitic burdens [73–75]. Low parasite load results in diminished coproantigen production and possible failure of the RDT to detect it [66, 72]. Conclusively, the sensitivity of this method implies that almost one out of six infected by *Giardia* spp. diarrheic dogs might test negative with the Speed^TM^ *Giardia* assay. To overcome it, it could be suggested for the practitioners either to repeat the test (after 48 h) or to test a pool sample from feces collected for two consecutive days in clinically suspected animals with a negative Speed^TM^ *Giardia* test result and, furthermore, in the case of dogs from the same breeding unit, it is recommended to conduct tests on several animals [63].

All samples of the group A (confirmed positive by the Speed^TM^ *Giardia* test) were tested positive with both the ProSpecT *Giardia* EZ Microplate Assay and PCR, resulting in 100% PPV. The specificity for *Giardia* spp. is also optimal, reaching 100% for RDT, as it has been extensively reported in the literature [64, 74–81] and confirmed by our study.

In the routine diagnostic practice, the veterinarian may face other possible combinations of contradictory results, as was the case in the present study. More precisely, seven specimens of group B were found positive according to ELISA, while at the same time they were negative by PCR. This suggests either an ELISA false positive or a PCR false negative result. In the first assumption, ELISA may indeed give a false positive result in a limited number of cases (2%), due to 98–100% specificity and positive predictive values (98–100%), as registered by the manufacturer.

In the case of the second assumption, PCR false negative results may arise because the DNA yields from feces remain poor [82, 83]. *Giardia* cysts wall is difficult to disrupt and this may lead to insufficient DNA yields, whereas at the same time stool specimens commonly contain compounds such as DNases, proteases, bile salts, and polysaccharides that might cause DNA degradation and inhibition of enzymatic reactions [56, 84]. In this study the QIAmp**®** Fast DNA Stool Mini Kit was used for DNA isolation, which provides high inhibitor removal efficiency. Additionally, a pre-treatment of the cysts with three freeze-thaw cycles and extension of the incubation time were incorporated in the protocol to maximize cyst lysis, which according to [85] renders the QIAmp**®** Fast DNA Stool Mini Kit more sensitive than other conventional extraction methods, i.e. the phenol-chloroform protocol. Another possible reason of failure to amplify *Giardia* spp. is the inhomogeneous distribution of the cysts within a sample [86], or other minor factors, which may be the case in our study. According to Rochelle et al. [87] PCR amplification of the *Giardia 18S* rRNA gene may result false negative due to the unusually high GC content of its sequence. However, the *18S* rRNA gene was selected as a target sequence in our study, because it is represented by high copy numbers (approximately 60 to 130 copies of *G. duodenalis* per nucleus, arranged in tandem repeats) and thus it is considered of higher sensitivity [88, 89]. Furthermore, to overcome the aforementioned GC limitation, the One *Taq®* 2× master mix with GC Buffer (M0483S; New England BioLabs Inc., Hitchin, UK) was used. This optimized blend of *Taq* and Deep Vent^TM^ DNA polymerases has higher fidelity than solely *Taq* and provides robust amplification of GC rich templates.

Another combination of contradictory results that arose was that 14 samples negative according to ELISA resulted positive to PCR. This implies either an ELISA false negative result or a PCR false positive one.

The ProSpecT *Giardia* EZ Microplate Assay has certain performance limitations and a false negative result is likely to occur when the antigen level in the sample is below the detection level of the assay [75]. Lower antigen levels may arise due to a low parasite load at the start of the infection and also in cases of some immunocompetent infected animals, which manage to maintain it very low, thus not detectable. Finally, since the ProSpecT^TM^ *Giardia* EZ Microplate Assay is an immunoassay commonly used for humans, the lower sensitivity of this assay in veterinary medicine could be attributed to genetic heterogeneity between *Giardia* spp. isolates of human and canine origin [90].

On the other hand, false positive PCR findings may occur due to excessive PCR cycling resulting in amplification of similar to the target sequence DNA, low specificity of the primers or through the inclusion of contaminated DNA within the reaction, either at the stage of DNA extraction or at the set-up process [91]. In this study a region of the *18S* rRNA was amplified by using a valid protocol, as described by Hopkins et al. [40], with well-tested cycling conditions and primers. Furthermore, all precautions regarding avoidance of contamination were taken, as proved by the inclusion of the negative control template which was similarly subjected to DNA extraction. Indeed, gel electrophoresis revealed that no contaminated nucleic acid was introduced in the master mix during specimen processing. All the above support the hypothesis that these fourteen samples were most likely ELISA false negative samples. In any case, a positive PCR test cannot discriminate living and dead protozoa, as genetic material is present in both cases. Consequently, although a positive PCR result indicates the detection of the pathogen, it cannot differentiate between its incidental presence and active infection with clinical manifestations. It is therefore evident that PCR results should be interpreted in conjunction with the case history and clinical evidence of giardiosis.

## Conclusions

Veterinary practitioners must be aware of canine giardiosis in order to take into appropriate account the impact of this underestimated protozoan infection in the canine population as well as its possible zoonotic implication. The present study contributes to the understanding of the complex diagnosis of canine giardiosis. To the best of our knowledge, this is the first attempt to evaluate the performance of the Speed^TM^ *Giardia* test, which is a very commonly used diagnostic approach in veterinary practice. The sensitivity of the test was sufficient while specificity was excellent for *Giardia* spp. This diagnostic tool supports valid sample testing that is more rapid, easy to use and interpret and affordable. In conclusion, the Speed^TM^ *Giardia* test can be a valuable tool in veterinary settings with a high caseload where rapid diagnosis is required as well as in smaller practices where other techniques are often not available or there is limited training in fecal flotation interpretation. Our findings highlight the need to further improve the quality of current diagnostic methods in terms of sensitivity. This may elucidate most of the diagnostic challenges and assist towards reliable surveys and effective treatment of giardiosis under the umbrella of one health leading to protection of animal and public health.

## Supplementary information

**Additional file 1: Table S1.** Excel dataset for 100 samples.

## Data Availability

The dataset supporting the conclusions of this study are included within the article and its Additional file 1.

## Abbreviations

DEPC: Diethyl pyrocarbonate
DFA: direct fluorescent antibody
ELISA: enzyme-linked immunosorbent assay
GSA 65: *Giardia* Specific antigen 65
IFA: immunofluorescence antibody
LR+: positive likelihood ratio
LR−: negative likelihood ratio
PCR: polymerase chain reaction
RDT: rapid diagnostic test
Sn: sensitivity
Sp: specificity
*18S* rRNA: *18S* ribosomal RNA

## References

[1] Tan L, Wu S, Abdullahi AY, Yu X, Hu W, Song M (2016). PCR-RFLP method to detect zoonotic and host-specific *Giardia duodenalis* assemblages in dog fecal samples. Parasitol Res..

[2] Thompson RCA (2000). Giardiasis as a re-emerging infectious disease and its zoonotic potential. Int J Parasitol..

[3] Adam RD (2001). Biology of *Giardia lamblia*. Clin Microbiol Rev..

[4] Mohamed AS, Glickman LT, Camp JW, Lund E, Moore GE (2013). Prevalence and risk factors for *Giardia* spp. infection in a large national sample of pet dogs visiting veterinary hospitals in the United States (2003–2009). Vet Parasitol..

[5] Painter JE, Gargano JW, Collier SA, Yoder JS (2015). Giardiasis surveillance—United States, 2011–2012. MMWR Suppl..

[6] Caccio SA, De Giacomo M, Pozio E (2002). Sequence analysis of the beta-giardin gene and development of a polymerase chain reaction-restriction fragment length polymorphism assay to genotype *Giardia duodenalis* cysts from human faecal samples. Int J Parasitol..

[7] Hunter PR, Thompson RCA (2005). The zoonotic transmission of *Giardia* and *Cryptosporidium*. Int J Parasitol..

[8] Mircean V, Gyorke A, Cozma V (2012). Prevalence and risk factors of *Giardia duodenalis* in dogs from Romania. Vet Parasitol..

[9] Ryan U, Caccio SM (2013). Zoonotic potential of *Giardia*. Int J Parasitol..

[10] Caccio SM, Ryan U (2008). Molecular epidemiology of giardiasis. Mol Biochem Parasitol..

[11] Heyworth MF (2016). *Giardia duodenalis* genetic assemblages and hosts. Parasite..

[12] Bouzid M, Steverding D, Tyler KM (2008). Detection and surveillance of waterborne protozoan parasites. Curr Opin Biotechnol..

[13] Plutzer J, Ongerth J, Karanis P (2010). *Giardia* taxonomy, phylogeny and epidemiology: facts and open questions. Int J Hyg Environ Health..

[14] Plutzer J, Torokne A, Karanis P (2010). Combination of ARAD microfibre filtration and LAMP methodology for simple, rapid and cost-effective detection of human pathogenic *Giardia duodenalis* and *Cryptosporidium* spp. in drinking water. Lett Appl Microbiol..

[15] Vanni I, Caccio SM, van Lith L, Lebbad M, Svard SG, Pozio E (2012). Detection of *Giardia duodenalis* assemblages A and B in human feces by simple, assemblage-specific PCR assays. PloS Negl Trop Dis..

[16] Caccio SM, Thompson RCA, McLauchlin J, Smith HV (2005). Unravelling *Cryptosporidium* and *Giardia* epidemiology. Trends Parasitol..

[17] Monis PT, Caccio SM, Thompson RCA (2009). Variation in *Giardia*: towards a taxonomic revision of the genus. Trends Parasitol..

[18] Thompson RCA (2004). The zoonotic significance and molecular epidemiology of *Giardia* and giardiasis. Vet Parasitol..

[19] Claerebout E, Casaert S, Dalemans AC, De Wilde N, Levecke B, Vercruysse J (2009). *Giardia* and other intestinal parasites in different dog populations in northern Belgium. Vet Parasitol..

[20] Cardoso AS, Costa IMH, Figueiredo C, Castro A, Conceicao MAP (2014). The occurrence of zoonotic parasites in rural dog populations from northern Portugal. J Helminthol..

[21] Pantchev N, Broglia A, Paoletti B, Vrhovec MG, Bertram A, Nockler K (2014). Occurrence and molecular typing of *Giardia* isolates in pet rabbits, chinchillas, guinea pigs and ferrets collected in Europe during 2006–2012. Vet Rec..

[22] Tan LP, Yu XG, Abdullahi AY, Wu S, Zheng GC, Hu W (2015). Development of a rapid HRM genotyping method for detection of dog-derived *Giardia lamblia*. Parasitol Res..

[23] Dorny P, Praet N, Deckers N, Gabriel S (2009). Emerging food-borne parasites. Vet Parasitol..

[24] Baldursson S, Karanis P (2011). Waterborne transmission of protozoan parasites: review of worldwide outbreaks—an update 2004–2010. Water Res..

[25] Feng YY, Xiao LH (2011). Zoonotic potential and molecular epidemiology of *Giardia* species and giardiasis. Clin Microbiol Rev..

[26] Popruk S, Thima K, Udonsom R, Rattaprasert P, Sukthana Y (2011). Does silent *Giardia* infection need any attention?. Open Trop Med J..

[27] Lane S, Lloyd D (2002). Current trends in research into the waterborne parasite *Giardia*. Crit Rev Microbiol..

[28] Bouzid M, Halal K, Jeffreys D, Hunter PR (2015). The prevalence of *Giardia* infection in dogs and cats, a systematic review and meta-analysis of prevalence studies from stool samples. Vet Parasitol..

[29] Symeonidou I, Gelasakis AI, Arsenopoulos KV, Schaper R, Papadopoulos E (2017). Regression models to assess the risk factors of canine gastrointestinal parasitism. Vet Parasitol..

[30] Barutzki D, Schaper R (2013). Age-dependant prevalence of endoparasites in young dogs and cats up to one year of age. Parasitol Res..

[31] Dubna S, Langrova I, Napravnik J, Jankovska I, Vadlejch J, Pekar S (2007). The prevalence of intestinal parasites in dogs from Prague, rural areas, and shelters of the Czech Republic. Vet Parasitol..

[32] Gal A, Harrus S, Arcoh I, Lavy E, Aizenberg I, Mekuzas-Yisaschar Y (2007). Coinfection with multiple tick-borne and intestinal parasites in a 6-week-old dog. Can Vet J..

[33] Caccio SM (2004). New methods for the diagnosis of *Cryptosporidium* and *Giardia*. Parassitologia..

[34] Garcia LS, Shum AC, Bruckner DA (1992). Evaluation of a new monoclonal-antibody combination reagent for direct fluorescence detection of *Giardia* cysts and *Cryptosporidium* oocysts in human fecal specimens. J Clin Microbiol..

[35] Rimhanen-Finne R, Enemark HL, Kolehmainen J, Toropainen P, Hanninen ML (2007). Evaluation of immunofluorescence microscopy and enzyme-linked immunosorbent assay in detection of *Cryptosporidium* and *Giardia* infections in asymptomatic dogs. Vet Parasitol..

[36] Rosoff JD, Sanders CA, Sonnad SS, Delay PR, Hadley WK, Vincenzi FF (1989). Stool diagnosis of giardiasis using a commercially available enzyme-immunoassay to detect giardia-specific antigen-65 (Gsa 65). J Clin Microbiol..

[37] Garcia LS, Shimizu RY (1997). Evaluation of nine immunoassay kits (enzyme immunoassay and direct fluorescence) for detection of *Giardia lamblia* and *Cryptosporidium parvum* in human fecal specimens. J Clin Microbiol..

[38] Uehlinger FD, Naqvi SA, Greenwood SJ, McClure JT, Conboy G, O’Handley R (2017). Comparison of five diagnostic tests for *Giardia duodenalis* in fecal samples from young dogs. Vet Parasitol..

[39] Koehler AV, Jex AR, Haydon SR, Stevens MA, Gasser RB (2014). *Giardia*/giardiasis—a perspective on diagnostic and analytical tools. Biotechnol Adv..

[40] Hopkins RM, Meloni BP, Groth DM, Wetherall JD, Reynoldson JA, Thompson RCA (1997). Ribosomal RNA sequencing reveals differences between the genotypes of *Giardia* isolates recovered from humans and dogs living in the same locality. J Parasitol..

[41] Berrilli F, Di Cave D, De Liberato C, Franco A, Scaramozzino P, Orecchia P (2004). Genotype characterisation of *Giardia duodenalis* isolates from domestic and farm animals by *SSU*-rRNA gene sequencing. Vet Parasitol..

[42] Ndao M (2009). Diagnosis of parasitic diseases: old and new approaches. Interdiscip Perspect Infect Dis..

[43] Ndao M (2010). Diagnosis of parasitic diseases: old and new approaches. Interdiscip Perspect Infect Dis..

[44] Faust E, Sawitz W, Tobie J, Odom V, Peres C (1938). Comparative efficiency of various technics for the diagnosis of protozoa and helminths in feces. J Parasitol..

[45] Taylor MA, Coop RL, Wall RL (2007). Veterinary parasitology.

[46] Foreyt W (2001). Veterinary parasitology: reference manual.

[47] Zajac AM, Conboy GA (2012). Veterinary clinical parasitology.

[48] Decock C, Cadiergues MC, Larcher M, Vermot S, Franc M (2003). Comparison of two techniques for diagnosis of giardiasis in dogs. Parasite..

[49] Cirak VY, Bauer C (2004). Comparison of conventional coproscopical methods and commercial coproantigen ELISA kits for the detection of *Giardia* and *Cryptosporidium* infections in dogs and cats. Berl Munch Tierarztl..

[50] Dryden MW, Payne PA, Smith V (2006). Accurate diagnosis of *Giardia* spp. and proper fecal examination procedures. Vet Ther..

[51] Geurden T, Berkvens D, Casaert S, Vercruysse J, Claerebout E (2008). A Bayesian evaluation of three diagnostic assays for the detection of *Giardia duodenalis* in symptomatic and asymptomatic dogs. Vet Parasitol..

[52] Traub RJ, Inpankaew T, Reid SA, Sutthikornchai C, Sukthana Y, Robertson ID (2009). Transmission cycles of *Giardia duodenalis* in dogs and humans in Temple communities in Bangkok—a critical evaluation of its prevalence using three diagnostic tests in the field in the absence of a gold standard. Acta Trop..

[53] Costa M, Clarke C, Mitchell S, Papasouliotis K (2016). Diagnostic accuracy of two point-of-care kits for the diagnosis of *Giardia* species infection in dogs. J Small Anim Pract..

[54] Uiterwijk M, Nijsse R, Kooyman FNJ, Wagenaar JA, Mughini-Gras L, Koop G, Ploeger HW (2018). Comparing four diagnostic tests for *Giardia duodenalis* in dogs using latent class analysis. Parasit Vectors..

[55] McGlade TR, Robertson ID, Elliot AD, Read C, Thompson RC (2003). Gastrointestinal parasites of domestic cats in Perth, Western Australia. Vet Parasitol..

[56] Scaramozzino P, Di Cave D, Berrilli F, D’Orazi C, Spaziani A, Mazzanti S (2009). A study of the prevalence and genotypes of *Giardia duodenalis* infecting kennelled dogs. Vet J..

[57] El-Badry A, Al-Ali A, Mahrous A (2010). Molecular identification & prevalence of *Giardia lamblia* & *Cryptosporidium* in duodenal aspirate in Al-Madinah. J Med Biomed Sci..

[58] Kehl KS, Cicirello H, Havens PL (1995). Comparison of four different methods for detection of *Cryptosporidium* species. J Clin Microbiol..

[59] Zimmerman SK, Needham CA (1995). Comparison of conventional stool concentration and preserved-smear methods with Merifluor *Cryptosporidium/Giardia* direct immunofluorescence assay and ProSpecT *Giardia* EZ Microplate Assay for detection of *Giardia lamblia*. J Clin Microbiol..

[60] Chapman PA, Rush BA, McLauchlin J (1990). An enzyme immunoassay for detecting *Cryptosporidium* in faecal and environmental samples. J Med Microbiol..

[61] Aldeen WE, Carroll K, Robison A, Morrison M, Hale D (1998). Comparison of nine commercially available enzyme-linked immunosorbent assays for detection of *Giardia lamblia* in fecal specimens. J Clin Microbiol..

[62] Maraha B, Buiting AGM (2000). Evaluation of four enzyme immunoassays for the detection of *Giardia lamblia* antigen in stool specimens. Eur J Clin Microbiol..

[63] Hanson KL, Cartwright CP (2001). Use of an enzyme immunoassay does not eliminate the need to analyze multiple stool specimens for sensitive detection of *Giardia lamblia*. J Clin Microbiol..

[64] Strand EA, Robertson LJ, Hanevik K, Alvsvag JO, Morch K, Langeland N (2008). Sensitivity of a *Giardia* antigen test in persistent giardiasis following an extensive outbreak. Clin Microbiol Infect..

[65] Mekaru SR, Marks SL, Felley AJ, Chouicha N, Kass PH (2007). Comparison of direct immunofluorescence, immunoassays, and fecal flotation for detection of *Cryptosporidium* spp. and *Giardia* spp. in naturally exposed cats in 4 northern California animal shelters. J Vet Intern Med..

[66] Rishniw M, Liotta J, Bellosa M, Bowman D, Simpson KW (2010). Comparison of 4 *Giardia* diagnostic tests in diagnosis of naturally acquired canine chronic subclinical giardiasis. J Vet Intern Med..

[67] Leib MS, Zajac AM (1999). Giardiasis in dogs and cats. Vet Pract..

[68] Saleh MN, Heptinstall JR, Johnson EM, Ballweber LR, Lindsay DS, Werre S, Herbein JF, Zajac AM (2019). Comparison of diagnostic techniques for detection of *Giardia duodenalis* in dogs and cats. J Vet Intern Med..

[69] Hooshyar H, Rostamkhani P, Arbabi M, Delavari M (2019). *Giardia lamblia* infection: review of current diagnostic strategies. Gastrenterol Hepatol Bed Bench..

[70] Uchoa FFM, Sudre AP, Macieira DB, Almosny NRP (2017). The influence of serial fecal sampling on the diagnosis of giardiasis in humans, dogs, and cats. Rev Inst Med Trop Sao Paulo..

[71] Uchoa FFD, Sudre AP, Campos SDE, Almosny NRP (2018). Assessment of the diagnostic performance of four methods for the detection of *Giardia duodenalis* in fecal samples from human, canine and feline carriers. J Microbiol Meth..

[72] Vidal AM, Catapani WR (2005). Enzyme-linked immunosorbent assay (ELISA) immunoassaying versus microscopy: advantages and drawbacks for diagnosing giardiasis. Sao Paulo Med J..

[73] Ali SA, Hill DR (2003). Giardia intestinalis. Curr Opin Infect Dis..

[74] Garcia LS, Shimizu RY, Novak S, Carroll M, Chan F (2003). Commercial assay for detection of *Giardia lamblia* and *Cryptosporidium parvum* antigens in human fecal specimens by rapid solid-phase qualitative immunochromatography. J Clin Microbiol..

[75] Johnston SP, Ballard MM, Beach MJ, Causer L, Wilkins PP. Evaluation of three commercial assays for detection of *Giardia* and *Cryptosporidium* organisms in fecal specimens. J Clin Microbiol. 2003;41:623–6. 10.1128/JCM.41.2.623-626.2003 PMC149727 12574257

[76] Sharp SE, Suarez CA, Duran Y, Poppiti RJ (2001). Evaluation of the triage micro parasite panel for detection of *Giardia lamblia*, *Entamoeba histolytica/Entamoeba dispar*, and *Cryptosporidium parvum* in patient stool specimens. J Clin Microbiol..

[77] Oster N, Gehrig-Feistel H, Jung H, Kammer J, McLean JE, Lanzer M (2006). Evaluation of the immunochromatographic CORIS *Giardia*-strip test for rapid diagnosis of *Giardia lamblia*. Eur J Clin Microbiol Infect Dis..

[78] Weitzel T, Dittrich S, Mohl I, Adusu E, Jelinek T (2006). Evaluation of seven commercial antigen detection tests for *Giardia* and *Cryptosporidium* in stool samples. Clin Microbiol Infect..

[79] Abdel Hameed DM, Elwakil HS, Ahmed MA (2008). A single-step immunochromatographic lateral-flow assay for detection of *Giardia lamblia* and *Cryptosporidium parvum* antigens in human fecal samples. J Egypt Soc Parasitol..

[80] Goni P, Martin B, Villacampa M, Garcia A, Seral C, Castillo FJ (2012). Evaluation of an immunochromatographic dip strip test for simultaneous detection of *Cryptosporidium* spp., Giardia duodenalis, and Entamoeba histolytica antigens in human faecal samples. Eur J Clin Microbiol Infect Dis..

[81] Minak J, Kabir M, Mahmud I, Liu Y, Liu L, Haque R (2012). Evaluation of rapid antigen point-of-care tests for detection of *Giardia* and *Cryptosporidium* species in human fecal specimens. J Clin Microbiol..

[82] Verweij JJ, Pit DSS, van Lieshout L, Baeta SM, Dery GD, Gasser RB (2001). Determining the prevalence of *Oesophagostomum bifurcum* and *Necator americanus* infections using specific PCR amplification of DNA from faecal samples. Trop Med Int Health..

[83] Nunes CM, Lima LG, Manoel CS, Pereira RN, Nakano MM, Garcia JF (2006). Fecal specimens preparation methods for PCR diagnosis of human taeniosis. Rev Inst Med Trop Sao Paulo..

[84] Nantavisai K, Mungthin M, Tan-ariya P, Rangsin R, Naaglor T, Leelayoova S (2007). Evaluation of the sensitivities of DNA extraction and PCR methods for detection of *Giardia duodenalis* in stool specimens. J Clin Microbiol..

[85] Babaei Z, Oormazdi H, Rezaie S, Rezaeian M, Razmjou E (2011). *Giardia intestinalis*: DNA extraction approaches to improve PCR results. Exp Parasitol..

[86] David EB, Coradi ST, Oliveira-Sequeira TCG, Ribolla PEM, Katagiri S, Guimaraes S (2011). Diagnosis of *Giardia* infections by PCR-based methods in children of an endemic area. J Venom Anim Toxins..

[87] Rochelle PA, De Leon R, Stewart MH, Wolfe RL (1997). Comparison of primers and optimization of PCR conditions for detection of *Cryptosporidium parvum* and *Giardia lamblia* in water. Appl Environ Microbiol..

[88] Boothroyd JC, Wang A, Campbell DA, Wang CC (1987). An unusually compact ribosomal DNA repeat in the protozoan *Giardia lamblia*. Nucleic Acids Res..

[89] Sil AK, Das P, Bhattacharyya S, Ghosh S, Chattopadhyay DJ (1998). Cloning of ribosomal RNA genes from an Indian isolate of *Giardia lamblia* and the use of intergenic nontranscribing spacer regions in the differentiation of *Giardia* from other enteric pathogens. J Biosci..

[90] Bianciardi P, Papini R, Giuliani G, Cardini G (2004). Prevalence of *Giardia* antigen in stool samples from dogs and cats. Revue Méd Vét..

[91] Lorenz TC (2012). Polymerase chain reaction: Basic protocol plus troubleshooting and optimization strategies. J Vis Exp..

